# Enhanced Antioxidant Activity of Ursolic Acid by Complexation with Copper (II): Experimental and Theoretical Study

**DOI:** 10.3390/ma14020264

**Published:** 2021-01-07

**Authors:** Mariola Samsonowicz, Monika Kalinowska, Kamila Gryko

**Affiliations:** Department of Chemistry, Biology and Biotechnology, Institute of Civil Engineering and Energetics, Faculty of Civil Engineering and Environmental Science, Bialystok University of Technology, Wiejska 45E Street, 15-351 Bialystok, Poland; m.kalinowska@pb.edu.pl (M.K.); k.gryko@pb.edu.pl (K.G.)

**Keywords:** ursolic acid, antioxidant, copper (II) complex, quantum-chemical calculations, spectroscopy

## Abstract

The copper (II) complex of ursolic acid (Cu(II) UA) was synthesized and discussed in terms of its infrared, UV–visible spectra, quantum-chemical calculations at B3LYP/6-31G(d) level and antioxidant capacity. The copper (II) complex was stable in methanolic solution with the molar ratio metal:ligand 1:1. The data obtained by FT-IR confirmed the metal ion coordination through the carboxylate anion. The antioxidant properties of ursolic acid and its complex with Cu were discussed on the basis of energy of the highest occupied molecular orbital (HOMO) and lowest unoccupied molecular orbital (LUMO) and values of chemical reactivity parameters. The antiradical properties of ursolic acid and the Cu (II) complex were examined against DPPH^•^ and HO^•^ radicals, and the ferric reducing antioxidant power (FRAP) was examined. The Cu(II) complex showed higher antioxidant activity than ursolic acid, i.e., in DPPH^•^ assay, the EC_50_ for UA was 47.0 mM, whereas, for Cu(II), UA EC_50_ = 19.5 mM; the FRAP value for UA was 20.8 µM_Fe2+_, and 35.4 µM_Fe2+_ for Cu(II) UA (compound concentration 3 mM). Although there was no distinct difference in the antioxidant activity against HO^•^ between these two chemicals, they were both better HO^•^ scavengers than DPPH^•^ and showed different kinetics in the reaction with DPPH^•^.

## 1. Introduction

**Figure 1:**
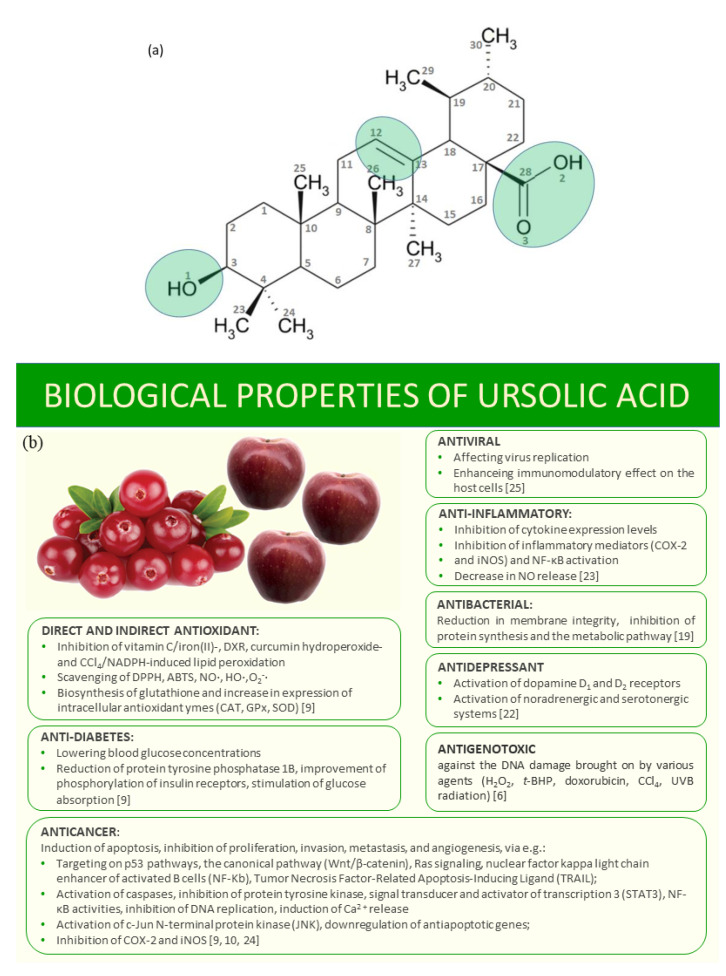
The scheme of ursolic acid, with marked active sites (**a**) and its biological properties (**b**).

Ursolic acid (3-beta-3-hydroxy-urs-12-ene-28-oic-acid; UA) (Figure 1a) is a plant triterpenoid compound found in some traditional medicinal herbs, fruits, vegetables and other plants, e.g., *V. macrocarpon* berries (0.460–1.090 g/kg fresh weight) [1], *Coffea arabica* coffee leaves (1.80 g/100 g dry weight) [2], *Malus domestica* apple pomace (0.80 g/100 g dry weight) [3], *Malus domestica* apple peels (1.351–0.324 mg/g dry weight) [4] and *Rosmarinus officinalis* rosemary leaves (2.95 g/100 g dry weight) [5]. Many studies have revealed that UA possesses anti-inflammatory, antiviral, hepatoprotective, immunomodulatory, antioxidant, gastroprotective, cardioprotective, vasculoprotective, neuroprotective and anticancer activities, combined with low toxicity (Figure 1b) [6,7,8]. The poor solubility of UA hinters its administration. The synthesis of new derivatives of ursolic acid or metal complexes may overcome this disadvantage and cause an increase in its therapeutic effects [9,10]. Moreover, the formulation of UA–phospholipid nanopowders with high bioavailability, targeting effect and stability was proposed [11]. Metal–ligand complexation is a well-known procedure to increase the solubility and biological activity of parent ligand. Tin, antimony and iron complexes with ursolic acid derivative showed better antibacterial properties against *Escherichia coli*, *Salmonella typhi*, *Shigella species*, *Staphylococcus aureus* and *Bacillus subtilis* and antioxidant activity in DPPH^•^ assay than ligand alone [12]. Similar results were obtained by Batool et al. [13]. Cu, Zn, Sn, Sb and Fe complexes of ursolic acid derivative containing aniline moiety revealed higher antifungal property toward *Tricophyton longifor*, *Fusarium solani*, *Bipol* and *Candida alibicans*; higher antibacterial activity against *Escherichia coli*, *Bacillus subtilis* and *Salmonella typhi*; and were better antioxidants (in DPPH^•^ test) than UA. The results pointed that metal complexation with ursolic acid may be a promising way to overcome its low solubility and may increase the biological application. There are many more examples of metal complexes with higher antioxidant or anticancer activities than ligand alone, e.g., alkali metal salts and zinc (II) complex of chlorogenic acid [14], Ca(II) complex of gentisic acid [15], metal complexes of cichoric acid [16] and metal complexes of flavones [17]. The reasons for increasing antioxidant activity of metal complexes compared to ligand alone include the following: (I) stabilization of the semiquinone radicals by metal cations, (II) involvement of the metallic center in the reaction with the radicals and acquisition of additional superoxide dismutating centers, (III) changes in the redox potential and thermochemical parameters (i.e., lower dissociation energy of O-H and ionization energy) of complexes compared to ligands, and (IV) different kinetics of the reaction between complexes/ligands and radicals. There are also many papers that describe the lowering of the antioxidant activity of ligands in the presence of metal cations [17]. Moreover, the chelation of metal ions (such as Fe or Cu) prevents metal-catalyzed free radical generation. Cu ions may convert the O_2_− and H_2_O_2_ into HO^•^ in Haber–Weiss reaction. The hydroxyl radicals are the most reactive oxygen species (the half-life in the range of 10^−9^ s), causing lipid peroxidation, DNA and protein damages in living cells [18].

As it was mentioned, ursolic acid shows wide biological properties, among other antioxidant activities [19]. Therefore, it can be a good radical scavenger, chain-breaking antioxidant or a chelator of radical-generating metals. It is well-known that many of the applied drugs cause harmful free-radical toxicity, including anticancer agents, non-steroidal anti-inflammatory drugs, antiretroviral agents, antipsychotics and analgesics. During the metabolism of these drugs, reactive intermediates may be produced that can reduce molecular oxygen directly to generate reactive oxygen species [20]. To overcome the severe toxic side effects, some well-known plant antioxidants may be applied in the treatment protocol. About 74% of the drugs approved by FDA are natural products or their derivatives [21]. Therefore, the studies on the mechanism of action of chemical compounds of natural origin are of great importance [22,23,24,25].

In this work, the antioxidant activity of ursolic acid (UA) was studied by means of ferric reducing power assay (FRAP), as well as in the reactions with DPPH^•^ (2,2-diphenyl-1-pikrylohydrazyl) and OH^•^ radicals. The copper (II) complex with ursolic acid was synthesized, and the effect of metal complexation on the antioxidant activity of ursolic acid was studied. The molecular structures of ursolic acid and the copper (II) complex (Cu (II) UA) were studied by means of quantum-chemical calculations, at the B3LYP/6-31G(d) level. The results are here discussed in terms of antioxidant properties of studied molecules.

## 2. Materials and Methods

### 2.1. Materials and Methods

#### 2.1.1. Materials

All chemicals were purchased from Sigma-Aldrich Co. (St. Louis, MO, USA) and used without purification. Only methanol was brought in POCH (Gliwice, Poland; http://www.poch.com.pl/). All solutions were prepared by using redistilled water.

#### 2.1.2. Preparation of Ursolic Acid–Cu (II) Complex (Cu (II) UA)

At first, a solution of the sodium salt of ursolic acid (UA) was prepared. The weighed mass of ursolic acid (0.0329 g) was added to 2 mL of methanolic solution of NaOH (0.036 M) in stoichiometric ratio 1:1. Then the mixed solution was placed in an ultrasonic bath, to completely dissolve the ursolic acid. Next, 2 mL of the aqueous solution of Cu (II) chloride (0.036 M) was added to the mixture (the molar ratio Cu:ligand was 1:1). The precipitate occurred immediately. It was washed several times with methanol. The correctness of the synthesis was discussed on the basis of the IR spectra recorded for solid (i.e., the disappearance of the band assigned to the stretching -C=O of the carboxylic group and appearance of the bands derived from stretching asymmetric and symmetric vibrations -COO- carboxylate anion coordinating metal ion). Moreover, the composition of the complex in methanolic solution was determined by the use of spectrophotometric titration. The spectrophotometric titration relied on adding increasing amounts of copper (II) chloride to 3 mL of the methanolic solution of sodium ursolate (0.05 mM) in ligand-to-metal molar ratios from 10 to 0.4 and monitoring the changes in absorbance at 266 nm. The stoichiometric composition of the complex was determined by molar ratio method.

#### 2.1.3. Instrumentation

The FT-IR spectra of the samples were recorded with an Alfa Bruker FT-IR spectrometer (Bremen, Germany) within the range of 400–4000 cm^−1^ with the resolution of 1 cm^−1^. The samples were measured in KBr matrix (the solid state of the samples) and in methanolic solution (the same solvent was used in antioxidant tests), using the ATR technique. The same optical range was measured. The effect of the KBr matrix and methanol on the sample was discussed. All spectrophotometric measurements in the range of UV–visible were done by the use of Agilent Carry 5000 spectrophotometer.

#### 2.1.4. Calculations

All calculations were carried out by using Gaussian 09W software package [26]. Visualization of calculated parameters was performed by GaussView 6 software (Gaussian Inc., Wallingford, CT, USA) [27]. Density Functional Theory (DFT) [28] was employed to get the optimized structure of complex at B3LYP level with 6–31G(d) basis set [29,30]. The calculated complex is cation with a 1+ charge, in which the presence of a counterion (chloride ion) was not included. The calculated structure was achieved with the absence of imaginary frequency. Determination of the highest occupied molecular orbital (HOMO) and lowest unoccupied molecular orbital (LUMO) energies was carried out by using the same level of theory. In addition, selected chemical reactivity parameters based on the HOMO–LUMO orbital’s energy were calculated [31,32].

### 2.2. Antioxidant Activity

#### 2.2.1. DPPH^•^ Antiradical Assay

The methanolic solution of DPPH^•^ at concentration 60 µM was prepared before the experiment. Ursolic acid and the copper (II) complex of ursolic acid were dissolved in methanol (C = 18 mM). The appropriate volumes of these solutions were added to 0.1 mL of DPPH^•^ and adjusted with methanol to the volume of 1 mL, so that the final concentrations of ursolic acid and its copper (II) complex were 16, 13, 10, 7 and 3 mM. The control probe consisted of only methanol an DPPH^•^. The samples were incubated through 1 h, and then the absorbance was read at the λ = 516 nm, using an Agilent Carry 5000 spectrophotometer. The percentage of inhibition of DPPH^•^ was calculated according to the following formula:

$$\%I=\frac{A_{control}-A_{sample}}{A_{control}}\cdot 100\%\;\left[\%\right]$$

where *A_control_* is the absorbance of the controls ample, and *A_sample_* is the absorbance of the tested sample. The EC_50_ values (the concentration of antioxidant that inhibits 50% of the initial concentration of DPPH^•^) were calculated on the basis of curve where the concentration of the tested substances was plotted versus the *%* inhibition of DPPH^•^. The kinetics of reaction between studied compounds and DPPH^•^ was studied. On the basis of the graph of DPPH^•^ concentration versus absorbance (y = 9.8796x + 0.0708; R² = 0.9996), the percentage of DPPH^•^ remaining after the reaction was calculated according to the following formula: $\%\;of\;remaining\;DPPH\cdot =\;\frac{A_{p}}{A_{0}}x100\%$, where *A_0_* is the absorbance of DPPH^•^· at the initial state, and *A_p_* is the absorbance of DPPH^•^ at each measuring point.

#### 2.2.2. HO^•^ Antiradical Assay

In total, 1 mL of ursolic acid or the copper complex of ursolic acid (C = 10, 7 and 3 mM), 300 µL FeSO_4_ (C = 8 mM), 1mL of salicylic acid (C = 3 mM) and 250 µL H_2_O_2_ (C = 20 mM) were mixed in a glass tube with a screw cap. The control sample was prepared in the same way, but instead of H_2_O_2_, the same volume of redistilled water was added. In blank sample, 1 mL of redistilled water was added instead of ursolic acid. The samples were incubated in a water bath, at 37 °C, during 30 min. Then, to each of the tested samples, 500 µL of redistilled water was added and placed in a centrifuge, for 5 min, at 3500 rpm/min. The absorbance of the samples was measured on a spectrophotometer, at a wavelength of λ = 510 nm. The scavenging activity of tested compounds was calculated according to the following formula:

$$\%I=\left\{1-\left[\frac{\left(A_{sample}-A_{control}\right)}{A_{blank}}\right]\right\}\cdot 100\%\;\left[\%\right]$$

where *A_sample_* is the absorbance of tested sample, *A_control_* is the absorbance of control sample and *A_blank_* is the absorbance of blank sample.

#### 2.2.3. Ferric-Reducing Power Assay (FRAP assay)

The 0.3 M acetate buffer (pH 3.6), 10 mM TPTZ (in 40 mM HCl) and 20 mM FeCl_3_·6H_2_O (in water) were mixed in a volumetric ratio 10:1:1 just before analyses. Then, 3 mL of this mixture was added to the tested substance (0.4 mL; C = 10, 7 and 3 mM). The absorbance was measured after 8 min of incubation at 594 nm, against a blank, using and Agilent Carry 5000 spectrophotometer. The antioxidant activity was expressed as Fe^2+^ equivalents (μM), using the calibration curve of FeSO_4_ (y = 54.46x–0.019; R^2^ = 0.996).

## 3. Results and Discussion

### 3.1. Quantum-Chemical Calculations

**Figure 2:**
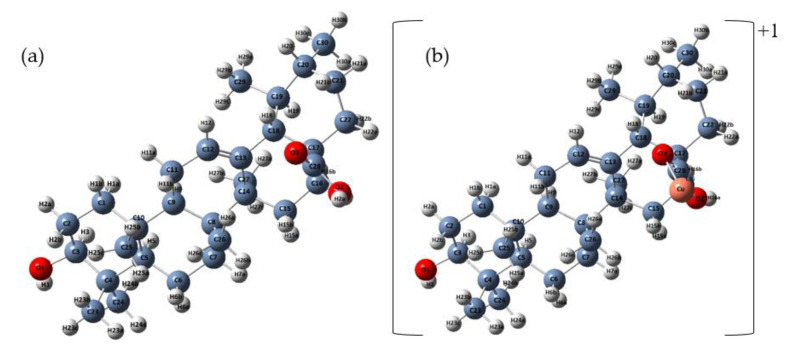
Optimized structures of ursolic acid (**a**) and cation (+1) complex of Cu (II) (**b**) calculated at B3LYP/6–31G (d) level.

The optimized structures at B3LYP/6–31G (d) basis set of ursolic acid and its complex cation (+1) of copper are presented in Figure 2. The initial structure for calculations was crystal structure of ursolic acid presented by Simon [33]. The calculated optimized geometrical parameters (bond lengths and bond angles) for ursolic acid and Cu (II) complex cation(+1) are gathered in Supplementary Materials Tables S1 and S2, respectively. The atom numbering of the ursolic acid molecule was shown in Figure 1a.

The experimental structure, bond lengths and angles of ursolic acid ethanol solvate reported by Simon et al. [33] (Supplementary Materials Tables S1 and S2) correlated well with those obtained in our calculations at B3LYP/6–31G(d) level; the correlation coefficients are R = 0.9687 and 0.8206, respectively. Larger differences between the theoretical parameters (bond lengths and angles) concerned mainly the region of the carboxyl and hydroxyl groups, which was probably caused by the influence of the ethanol solvent.

Table 1 summarized the structural parameters that changed in length by more than 0.005 Å. When analyzing the influence of the metal cation on the structure of the ursolic acid molecule, we noted that the most pronounced changes were observed in the geometry of carboxylic group. Coordination of Cu(II) through the carboxylate group caused the extension of the C28–O2 bond and the shortening of the C28–O2 bond; as a result, the lengths of these bonds became almost equal. The bond length between the O2–H2A/Cu atoms in formed cation (+1) complex increased in comparison to the ligand molecule, from 0.976 to 1.898 Å. The Cu^2+^ ion affected not only the geometry of the group C17C28O2O3^−^, but also the length of some bonds in the rings E (C17–C22, C17–C18 and C19–C18 bonds increased), D (C14–C13; C13–C18 bonds decreased) and C (the length of the double bond between C12 = C13 atoms increased).

When we analyzed the size of values of angles, the greatest changes for C28–O2–H2a/Cu (the increase by 30.71°) and C28–O3–H2a/Cu (the decrease by 18.54°) of carboxylate group, in comparison to the acid molecule, were observed. For other angles, a slight increase or decrease in their size by about 1–2° for some angles in the E, D and C rings (see Figure 1) was noticed. Table 2 lists only those angles for which the changes were greater than 1°after the formation of the complex cation.

**Figure 3:**
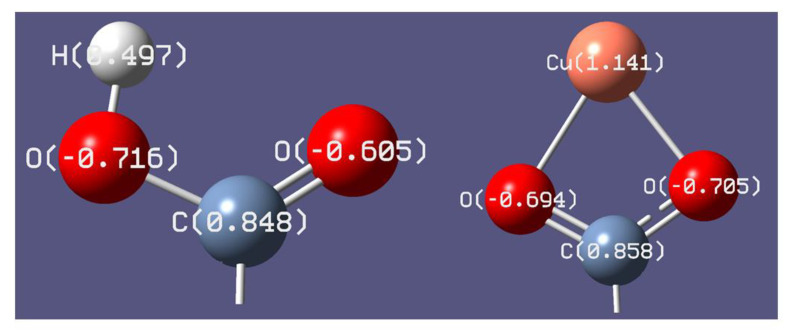
The distribution of NBO atomic charges in the carboxyl group in ursolic acid and carboxylate anion in metal complex calculated at B3LYP/6-31G(d) level.

NBO (natural bond orbital) atomic charges gathered on atoms in studied molecules were presented in Supplementary Materials Table S3. The formation of a complex cation (+1) with copper (II) caused a change in the negative charge: an increase in the charge on the O3 atom (by 0.1) and a decrease in the charge on the O2 atom (by 0.022) in the -COO^−^ group (Figure 3).

**Figure 4:**
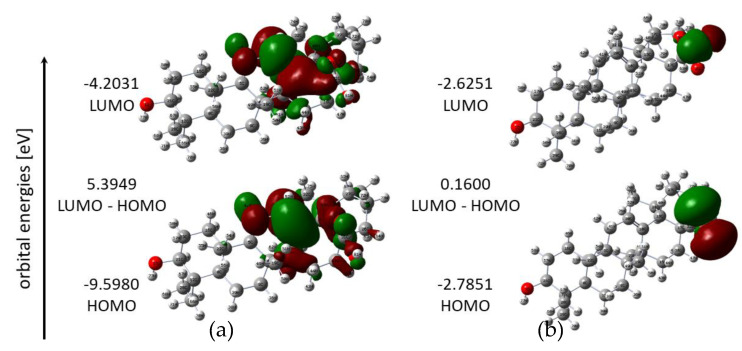
HOMO and LUMO energy orbitals of UA (**a**) and Cu(II) UA (**b**).

The energies of HOMO and LUMO orbitals are important parameters in quantum chemical calculations and very useful to qualitative descriptions of biological activity and molecule reactivity [34]. The energy of HOMO characterizes the electron-donating ability of a molecule, while LUMO energy determines the ability to accept an electron. The HOMO energy is an important electronic parameter for describing the antioxidant ability because it can be related to electron-transfer reactions. The antioxidative properties of many antioxidants can be elucidated by the distribution of the HOMO molecular orbitals and their energy values obtained with quantum mechanical calculations. The antioxidants with lower energy of HOMO orbital are less able to donate electrons during interactions with free radicals [35]. The higher the HOMO energy, the higher the capacity to realize nucleophilic attacks [16,36]. The HOMO and LUMO maps of ursolic acid and its complex with Cu molecules are shown in Figure 4. The HOMO orbital was mostly distributed over the double bond (C10–C20) and over six-membered ring (ring II) and peripheral functional groups in the acid. Meanwhile, the HOMO orbital in the complex was distributed over the carboxylic group and the metal ion. In the acid, the LUMO orbital was localized in the same regions as HOMO, which may indicate that these regions were chemically active, taking into account the frontier molecular orbital theory. The LUMO region in Cu(II) UA was concentrated on metal ions. The HOMO value for UA was −6.0505 eV and it was lower than for Cu (II) UA complex (−4.7000 eV).

The energy difference between LUMO and HOMO orbitals (the energy gap) is important for stability of structure and explains the eventual charge transfer interaction within the molecule, which in turn affects its biological activity. A molecule with a small energy gap is characterized by greater bioactivity, high chemical reactivity and low kinetic stability. As it is seen from Table 3 and Figure 4, the metal complex had a low value of energy gap, much smaller in comparison with acid molecule. It means that the electron can be easily transferred from HOMO to LUMO in Cu (II) complex. Therefore, Cu (II) UA were more reactive than UA.

The HOMO and LUMO energy values were used to calculate global reactive descriptors [31], using the equations given in Table 3. The HOMO energy is related to the ionization potential (I = -E_HOMO_). In general, the higher the HOMO energy of a molecule, the better its properties as an electron donor, and the lower the ionization potential, the lower the energy needed to remove an electron. According to the calculations, the copper (II) complex has lower ionization potential (2.7851 eV) and higher energy of HOMO orbital (−2.7851 eV), which indicates that it has a stronger electron-donating ability when compared to ursolic acid. The LUMO energy corresponds to the electron affinity (A = -E_LUMO_). The higher the electron affinity of a molecule and electronegativity, the better its properties as an electron acceptor, so UA is the better electron acceptor than Cu (II) UA. Chemical softness and hardness are properties of molecules related to their chemical reactivity. Cu (II) UA had a higher chemical softness and lower hardness than UA, indicating its higher chemical reactivity and lower stability. It may explain the higher antioxidant activity of copper ursolate because of its greater ability to donate electrons, as compared with acid alone.

**Figure 5:**
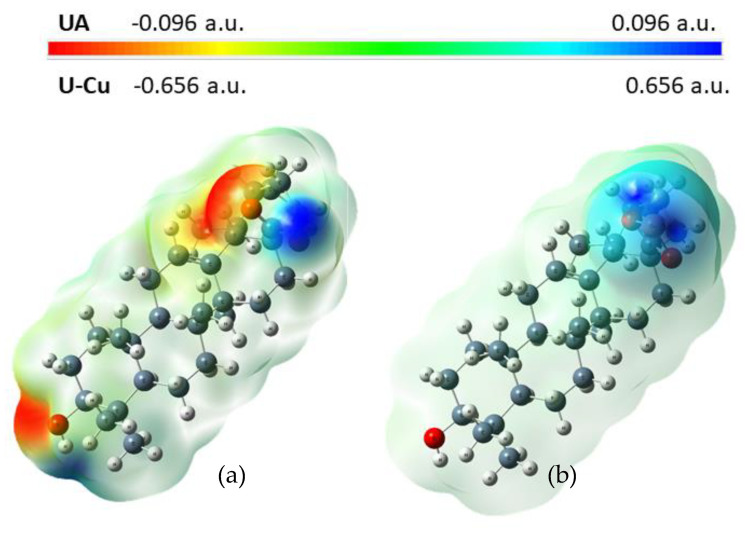
Molecular electrostatic potential map (MEP) for UA (**a**) and Cu (II) UA (**b**).

Molecular electrostatic potential is widely used as a reactivity map, representing the most likely region for nucleophilic and electrophilic attacks. The negative molecular electrostatic potential (MEP) (red color) corresponds to the regions related to electrophilic reactivity. The positive (blue color) regions of MEP are related to nucleophilic reactivity. Potential decreases are in the order of blue > green > yellow > orange > red [37]. A molecular electrostatic potential map of ursolic acid and its complex cation (+1) with copper (II) is presented at Figure 5. The electrostatic potential map of ursolic acid shows the carbonyl and hydroxyl group and carbon–carbon π bonds as preferred active sites for electrophilic attack. On the other hand, oxygen (O2) from the carboxyl group is a favorable site for electrophilic attack of Cu^2+^. This is confirmed by the FTIR analysis of ursolic acid and its copper (II) complex. In the spectrum of the salt, the bands from the carboxylate group disappeared (e.g., ν(C=O) at 1715 cm^−1^), and new ones from the carboxylate anion appeared (ν_as_(COO^−^) at 1541 cm^−1^; ν_s_(COO^−^) at 1402 cm^−1^).

### 3.2. UV Spectra

**Figure 6:**
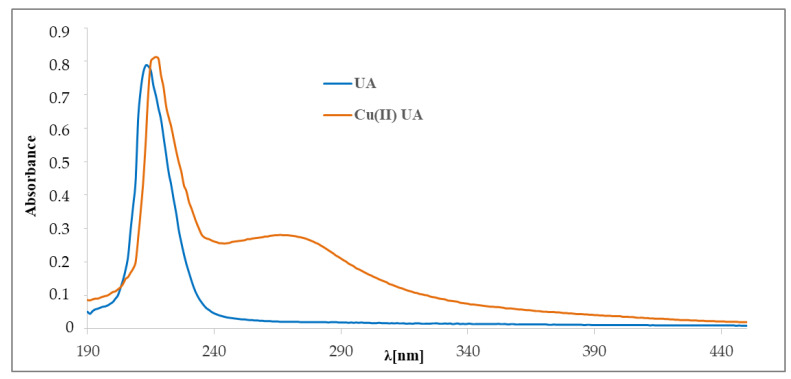
The UV spectra of ursolic acid and Cu (II) UA complex (0.05 mM).

The UV spectra of UA and its complex with Cu (II) were registered in methanol and are presented in Figure 6. In the UV spectrum of UA, we observed one maximum absorbance at 215 nm assigned to the π→π * transition within the carbon C_12_ = C_13_ in ring. The maxima of the bands in Cu (II) UA were located at 218.0 and 266.0 nm. The absorption maximum appearing in acid at 215 nm was slightly shifted (after complex formation) towards higher wavelengths (218 nm).

**Figure 7:**
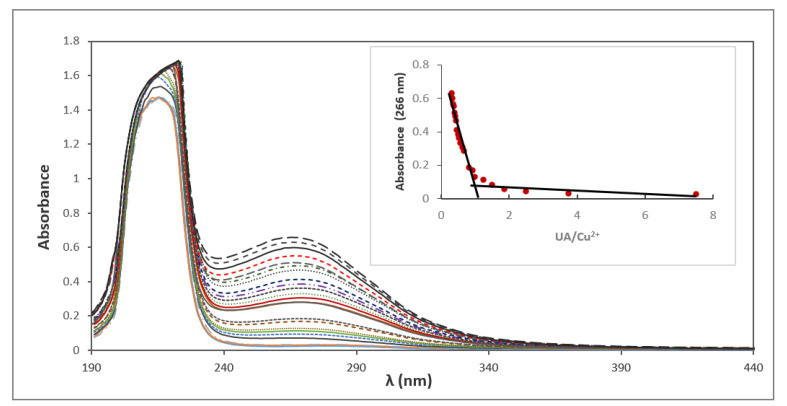
Spectrophotometric titration of methanolic solution of ursolic acid by Cu (II). Inset: complex absorbance at 266 nm versus [UA]/[Cu (II)].

To study of the composition of the complex Cu (II) UA in solution, spectrophotometric titration and the molar ratio method were applied. Absorption spectra of ursolic acid in methanolic solutions of increasing concentration of CuCl_2_ are shown in Figure 7. The absorbance and the position of the band at 215 nm was slightly shifted. At 266 nm, a new band appeared—the absorbance increased with the amount of added CuCl_2_. The molar ratio plot indicated the formation in solution of an ursolic acid–Cu (II) complex with a molar ratio of 1:1 (Figure 7, inset).

### 3.3. Vibrational Spectra

**Figure 8:**
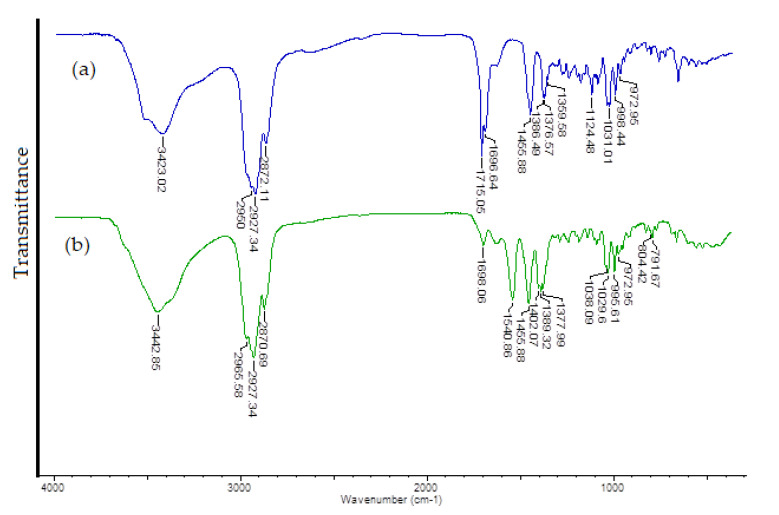
The FTIR spectra of ursolic acid (**a**) and Cu (II) complex of ursolic acid (**b**) registered for solid samples in the KBr matrix pellet.

The FTIR spectra of ursolic acid and its Cu (II) complex were recorded and the spectral data were gathered in Table 4. The Figure 8 showed the FTIR (KBr) spectra of UA and Cu (II) UA. Interpretation and assignment of bands was done on the basis of the literature works concerning ursolic acid and on our personal experience [38,39].

The ursolic acid spectrum included a characteristic band connected with the stretching vibrations of the carboxyl group: a very intense band at 1715 cm^−1^. In the FTIR spectra of acid, several bands derived from the vibrations of the hydroxyl groups were present. These bands of medium intensity were located at stretching vibrations (νOH) 3423 cm^−1^ (KBr) and 3402 cm^−1^ (ATR) and deformations (symmetrical and asymmetrical; βOH) at 1456 and 1377 (KBr) and 1452 (ATR) cm^−1^. By comparing the FTIR spectra of ursolic acid and its Cu (II) complex, it can observed that same characteristic bonds present in the spectrum of the acid were absent in the spectrum of the complex. The most important differences were the disappearance of the band at 1715 cm^−1^ (derived from the stretches of the C=O) and the appearance of new bands corresponding to vibrations of the carboxylate anion at 1541 and 1402 cm^−1^. These changes indicated the coordination of the metal ion through the carboxylate anion.

### 3.4. Antioxidant Tests

#### 3.4.1. Antiradical DPPH^•^ and HO^•^ Activity

**Figure 9:**
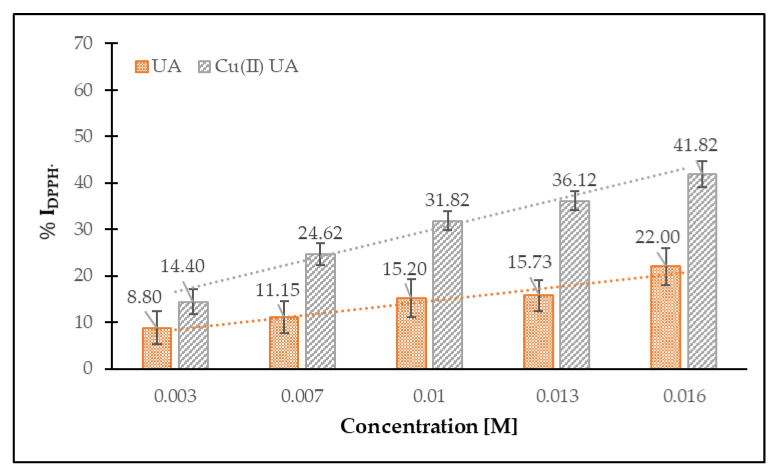
Percent of inhibition of DPPH^•^ radicals by ursolic acid (UA) and Cu (II) complex of ursolic acid (Cu (II) UA), depending on their concentration (0.003–0.016 M).

The antiradical DPPH^•^ activity of studied compounds was expressed as a percent of inhibition of the radical, % I DPPH^•^ (Figure 9). Cu (II) complex of ursolic acid showed better antiradical activity against DPPH^•^ than the ligand alone. With the increase in the concentration of the studied compounds, their antiradical properties increased as well as. In the range of concentration from 0.003 to 0.016 M the Cu (II) complex inhibited 14.40–41.82% of the initial concentration of DPPH^•^, whereas ursolic acid inhibited 8.80–22.00% (Figure 9). The EC_50_ value for UA was 47.0 mM, whereas, for Cu (II), the UA EC_50_ = 19.5 mM. In the literature, different values of the EC_50_ for UA were reported: 0.108 mM [40], 0.131 mM [41] and 6.88 µM [42]; for the concentration of UA, 0.333 mM the % I = 10.62 [43]. The obtained values of EC_50_ for the studied compounds pointed that the studied compounds were rather week scavengers of the radicals, when compared with some phenolic compounds or commercially applied antioxidants (butylated hydroxytoluene or butylated hydroxyanisole) [14,15]. On the other hand, it is interesting that the complexation of Cu^2+^ with ursolic acid caused an increase in the antiradical activity of the molecule, as compared with ligand alone. It was confirmed by other authors. A great number of Cu (II) complexes with non-steroidal anti-inflammatory drugs that possessed higher-than-ligand antiradical activity against DPPH^•^, hydroxyl and ABTS radicals was described in Reference [44]. Copper (II) complexes containing the tetradentate ligand 2-{[(3-chloro-2-hydroxy-propyl)-pyridin-2-ylmethyl-amino]-methyl}-phenol showed activity as superoxide dismutase or catalase mimetics and decreased ROS (reactive oxygen species) levels in glioma cells [45]. The antioxidant properties of Cu (II) complexes with curcumin, evaluated by the use of the photochemiluminescence method, were higher than for curcumin [46]. The higher activity of Cu (II) complexes, as compared with ligand alone, was mainly explained by (a) the stabilization of the semiquinone radicals by metal cations, (b) the redox reactions within the Cu^2+^/Cu^+^ couple (especially in the case of di- or multinuclear complexes) or (c) different reaction kinetics for ligand and complex.

**Figure 10:**
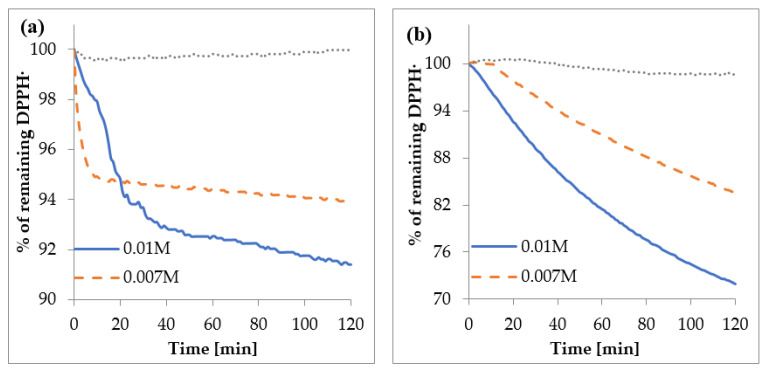
The percent of remaining DPPH^•^ vs. time in the presence of (**a**) UA and (**b**) Cu (II) UA.

Different factors may affect the course of reaction between antioxidants and DPPH^•^. The structure of antioxidants, pH and type of solvent determine the kinetics of the reaction. Some of them act as a fast antioxidant (the reaction with DPPH^•^ lasts even several seconds), or the equilibrium is reached after longer period of time (even over an hour). The schemes showing the remaining concentration of DPPH^•^ radicals in reaction mixture versus time of reaction are shown in Figure 10. The compounds were tested for the range of concentration: 0.003–0.016 M. The lowest concentration showed no effect on scavenging of the DPPH^•^ radicals. Then the % of remaining DPPH^•^ was lowering in a concentration-dependent manner. The higher the concentration of compounds, the lower amount of remaining DPPH^•^ radicals in a reaction mixture. In the case of UA, the reaction with DPPH^•^ had a biphasic character. The kinetic curve showed rather fast and slow stages. The highest rate of DPPH^•^ decay occurred during the first 10–30 min of the reaction, but the steady state was not reached until the end of the experiment, i.e., 120 min. In the case of Cu (II) UA, the kinetics curve only contained a slow-kinetics stage, and the Cu (II) complex maintained the antioxidant effect throughout the whole experiment (120 min). Antioxidants can react with DPPH^•^ via electron (SET) or hydrogen atom transfer (HAT) mechanisms (or the combination of them). Because the electron transfer mechanism is very fast (even femtoseconds), and the hydrogen atom transfer is slower, the first stage of quenching DPPH^•^ by UA may be described as a hydrogen atom transfer or mixed mechanism, even if the electron transfer mechanism is favorable over hydrogen transfer in methanol solution [47]. The next slow range of reaction between DPPH^•^ and UA may be explained by occurrence of secondary reactions (dimerization or disproportionation) [48]. On the other hand, the kinetic curve of the reaction of DPPH^•^ with Cu (II) showed only a slow-kinetics stage, which corresponds to the reversible process of radicals quenching and the secondary reactions [49]. The slow reaction of UA and Cu (II) UA with DPPH^•^ radicals may be explained by steric hindrance, as well as by obstructed access to DPPH^•^.

**Figure 11:**
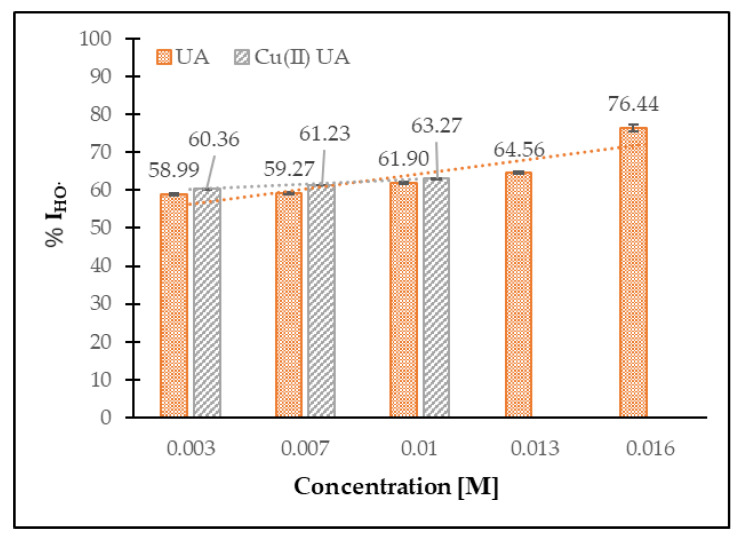
The percentage of HO^•^ inhibition by UA and Cu (II) UA at the concentration range of 0.003–0.016 M.

In Figure 11, the percentage of HO^•^ inhibition by UA and Cu (II) UA at the concentration range of 0.003–0.016 M is depicted. The compounds possessed antiradical activity toward hydroxyl radicals; ~60% of the initial concentration of HO^•^ was decayed by UA and its Cu (II) complex, in a similar manner. With the increase in the concentration of UA, the antiradical activity increased as well. UA and Cu (II) UA were better scavengers of HO^•^ than DPPH^•^.

#### 3.4.2. Ferric-Reducing Antioxidant Power (FRAP Assay)

**Figure 12:**
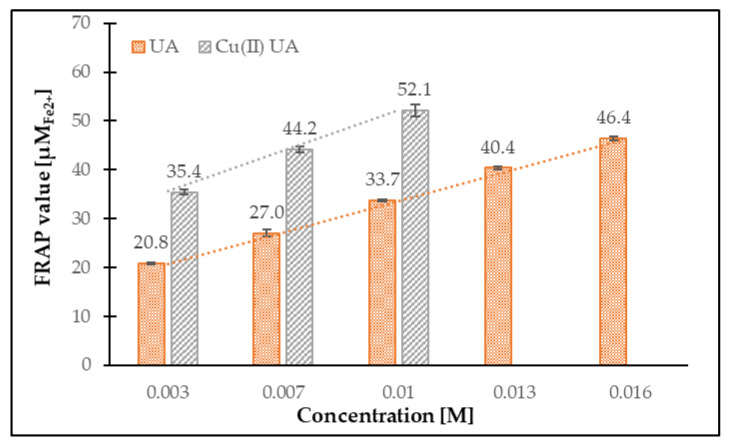
The ferric-reducing antioxidant activity of UA and Cu (II) UA in the concentration range 0.003–0.016 M.

The FRAP values obtained for UA and Cu (II) UA are shown in Figure 12. Cu (II) UA had higher ferric-reducing power, compared with ligand alone, and the activity increased with the raising concentration of the compounds. The FRAP assay is based on the reduction of ferric-tripyridyltriazine (Fe^3+^-TPTZ) to ferrous-tripyridyltriazine (Fe^2+^-TPTZ) by antioxidant. Therefore, the assay measures the ability of transfer an electron from the antioxidant to a compound, in order to reduce it. The mechanism is called single electron transfer (SET), and ionization potential (IP) is an important parameter that describes the antioxidant potential of the compounds. The calculated IP for UA was 9.5980 eV, whereas, for the Cu (II) complex, IP = 2.7851 eV. The higher antioxidant property of Cu (II) UA, as compared with UA, may be caused by easier electron abstraction from covalently bound ligand to metal cation than ligand alone. The additional parameters that describe the compounds with antioxidant properties are energy gap value (ΔE = E_LUMO_-E_HOMO_) and softness (S). The calculated softness for UA was 0.1854 eV, whereas, for the Cu (II) complex, S = 6.2499 eV. The softness describes the ability of a molecule to undergo deformation, and the higher the softness, the lower the energy necessary for the transfer of electron from HOMO to LUMO level, what probably generates more stable radicals after the donation of electrons. The calculated energy gap was greatly lower for Cu (II) UA (ΔE = 0.1600 EV) than UA (ΔE = 5.3949 eV), pointing at the higher reactivity of the Cu (II) complex and easy electronic transition.

## 4. Conclusions

Ursolic acid, a pentacyclic triterpenoid identified in the skin of apples, evokes a special interest because of its biological activity and possible application in pharmacy and medicine. The complexation with metal ions is the common way to improve the physicochemical properties of ligands, i.e., solubility, lipophilicity, stability and reactivity, among other antioxidant capacities. The studies revealed that the antioxidant activity of UA significantly increased after complexation with Cu (II), to a greater extend against HO^•^ than DPPH^•^ radicals. The kinetics reaction between ligand and its metal complex may differ significantly. The quantum chemical calculations at the B3LYP/6-31G(d) level predicted the geometries of UA and the reactivity of studied molecules well. The calculations were made for the complex with a metal:ligand ratio of 1:1, according to the results of the spectrophotometric analysis. The application of many analytical methods, combined with the quantum-chemical calculations gave accurate information about the effect of metal ions on the electronic charge of molecule and allowed for discussion of the changes in biological properties and reactivity of ligand upon metal complexation.

## Figures and Tables

**Table 1:** The selected bond lengths in ursolic acid and cation (+1) complex of Cu(II) molecules calculated by using B3LYP/6-31G(d) level.

Atom Numbers *	UA	Cu (II) UA
	Distances Between Atoms (Å)	
O2–H2a/Cu	0.976	1.898
O2–C28	1.361	1.285
C28–O3	1.213	1.293
O3–H2a/Cu	2.247	1.886
C28–C17	1.533	1.508
C17–C22	1.566	1.574
C19–C18	1.565	1.578
C18–C17	1.561	1.568
C14–C13	1.540	1.532
C13–C18	1.537	1.522
C11–C12	1.501	1.488
C12–C13	1.340	1.363

* Atom numbers according to Figure 1.

**Table 2:** The selected angles (°) between bonds in ursolic acid and cation (+1) complex of Cu (II) molecules calculated by using B3LYP/6-31G(d) level.

Angles	UA	Cu (II) UA
O3–C28–O2	120.95	115.48
C28–O2–H2a/Cu	105.46	86.92
C28–O3–H2a/Cu	56.50	87.21
O2–H2a–O3	77.09	70.38
C28–C17–C22	105.86	104.83
O3–C28–C17	126.15	121.59
O2–C28–C17	112.79	122.83
C17–C22–H22a	108.30	107.01
C17–C18–H18	106.47	107.60
H19–C19–C18	108.82	107.66
C13–C14–C27	108.38	107.17
C18–C13–C14	121.15	122.28
C12–C11–C9	113.11	114.12
C8–C14–C27	112.11	110.46

**Table 3:** Chemical reactivity parameters calculated for UA and Cu (II) UA

Parameter	UA	Cu (II) UA
Energy (Hartree *)	−1397.7661	−3037.1805
Dipole moment (Debye)	2.5264	13.8294
E_HOMO_ (eV)	−9.5980	−2.7851
E_LUMO_ (eV)	−4.2031	−2.6251
Energy gap (eV)	5.3949	0.1600
Ionization potential, I = −E_HOMO_ (eV)	9.5980	2.7851
Electronaffinity, A = −E_LUMO_ (eV)	4.2031	2.6251
Electronegativity, $\chi =\frac{I+A}{2}$ (eV)	6.9005	2.7051
Electronic chemical potential, $\mu =-\frac{I+A}{2}$ (eV)	−6.9005	−2.7051
Chemical hardness, $\eta =\frac{I-A}{2}$ (eV)	2.6975	0.0800
Chemical softness, S $=\frac{1}{2}$ (eV)	0.1854	6.2499
Electrophilicity index, $\omega =\frac{\mu^{2}}{2}$ (eV)	8.8263	45.7334

* 1 Hartree = 2625.5 kJ/mol.

**Table 4:** Wavenumbers (cm^−1^), intensities and assignments of the selected bonds occurring in the FTIR spectra of ursolic acid (UA) and its Cu (II) complex.

UA		Cu (II) UA Complex		Assignments
IR KBr	IR–ATR	IR KBr	IR–ATR	
3423 s	3402w	3443 s		ν (OH)
-		2966 s		ν(OH)
2950 vs	2948 vs	-		ν (CH)
2927vs	2926 vs	2927 vs		ν(CH)
2872 s	2835 s	2871 s	2844vw	ν(CH)
1715 s		-		ν(C=O)
1697 s	1659 w	1698 w	1636w	ν(C=O)
-		1541 m		ν_as_(COO^-^)
1456 m	1452 m	1456 s	1454 w	β_as_ (OH)
-		1402 m		ν_s_(COO^−^)
1386 m		1389 m	1387w	δ_s_(CH_3_)
1377 m		1378 m		β_s_(OH)
1360 w		-		ν(C-OH)
1031 m	1029 m	1030 m		(C-OH)
998 m		996 m		γ(CH) _– C = C −_
973 w		973 w		ν (C-C), C-H, C-O
-		804 w		δ_s_(CH_3_)

## Data Availability

The data presented in this study are available on request from the corresponding author.

## Supplementary Materials

The following are available online at https://www.mdpi.com/1996-1944/14/2/264/s1. Table S1: The bond lengths of ursolic acid and cation (+1) complex of Cu (II) molecules calculated by using B3LYP/6-31G(d) *. Table S2: The bond angles of ursolic acid and cation (+1) complex of Cu (II) molecules calculated by using B3LYP/6-31G(d) *. Table S3: Data of natural bond orbital (NBO) atomic charge analysis for ursolic acid and cation (+1) complex of Cu (II).
