# Supplementary material for: Enhanced Antioxidant Activity of Ursolic Acid by Complexation with Copper (II): Experimental and Theoretical Study
Source: Materials (Basel). 2021 Jan 7;14(2):264. doi: 10.3390/ma14020264 (PMC7825779; doi:10.3390/ma14020264)
Supplement: Supplementary file 1 [file materials-14-00264-s001.pdf]

materials

MDPI

Supplementary Materials

Enhanced Antioxidant Activity of Ursolic Acid by Complexation with Copper (II): Experimental and Theoretical Study

Mariola Samsonowicz *, Monika Kalinowska and Kamila Gryko

Department of Chemistry, Biology and Biotechnology, Institute of Civil Engineering and Energetics, Faculty of Civil Engineering and Environmental Science, Bialystok University of Technology, Wiejska 45E Street, 15-351 Bialystok, Poland; m.kalinowska@pb.edu.pl (M.K.), k.gryko@pb.edu.pl (K.G.)
* Correspondence: m.samsonowicz@pb.edu.pl

Table S1. The bond lengths of ursolic acid and cation (+1) complex of Cu(II) molecules calculated using B3LYP/6-31G(d)*.

	Bond lengths (Å)		
	**UA**	**UA exp.[33]**	**Cu(II) UA**
O2–H2a	0.976	0.804	1.898
O2–C28	1.361	1.183	1.285
C28–O3	1.213	1.316	1.293
O3–H2a	2.247	2.822	1.886
		E - ring	
C28–C17	1.533	1.525	1.508
C17–C22	1.566	1.552	1.574
C22–C21	1.528	1.513	1.527
C21–C20	1.550	1.503	1.549
C20–C19	1.563	1.568	1.561
C19–C18	1.565	1.547	1.578
C18–C17	1.561	1.533	1.568
C20–C33	1.537	1.523	1.538
C19–C29	1.534	1.524	1.534
C22–H22a	1.096	1.032	1.097
C22–H22b	1.098	0.775	1.096
C21–H21a	1.097	1.023	1.097
C21–H21b	1.097	1.120	1.096
C20–H20	1.100	0.925	1.099
C30–H30a	1.097	1.036	1.095
C30–H30b	1.098	1.022	1.098
C30–H30c	1.096	0.962	1.095
C19–H19	1.100	1.104	1.098
C29–H29a	1.097	0.993	1.096
C29–H29b	1.096	0.983	1.094
C29–H29c	1.093	0.888	1.093
C18–H18	1.095	1.055	1.097
		D – ring	
C17–C16	1.543	1.517	1.541
C16–C15	1.529	1.521	1.529
C15–C14	1.558	1.541	1.558
C14–C13	1.540	1.532	1.532

C13–C16	1.537	1.529	1.522
C16–H16a	1.095	0.968	1.095
C16–H16b	1.097	0.734	1.095
C15–H15a	1.097	1.020	1.095
C15–H15b	1.093	0.888	1.093
C27–H27a	1.092	0.902	1.092
C27–H27b	1.095	1.112	1.093
C27–H27c	1.094	1.098	1.092
C14–C27	1.557	1.563	1.562
C – ring			
C14–C8	1.608	1.581	1.6110
C8–C9	1.570	1.558	1.5696
C9–C11	1.538	1.532	1.5373
C11–C12	1.501	1.510	1.4881
C12–C13	1.340	1.314	1.3625
C8–C26	1.553	1.565	1.5517
C26–H26a	1.090	1.048	1.0887
C26–H26b	1.094	0.987	1.0940
C26–H26c	1.094	0.987	1.0967
C9–H9	1.098	0.924	1.0966
C11–H11a	1.100	1.101	1.1017
C11–H11b	1.098	0.997	1.0992
C12–H12	1.090	0.926	1.0900
B – ring			
C4–C7	1.551	1.529	1.551
C7–C6	1.534	1.521	1.536
C6–C5	1.539	1.522	1.539
C5–6C	1.570	1.551	1.569
C10–C9	1.588	1.582	1.586
C7–H7a	1.098	1.030	1.098
C7–H7b	1.095	0.908	1.095
C6–H6a	1.095	0.929	1.094
C6–H6b	1.096	0.945	1.096
C5–H5	1.104	1.050	1.104
C25–H25a	1.093	1.056	1.094
C25–H25b	1.092	0.878	1.091
C25–H25c	1.092	0.982	1.092
C10–C25	1.552	1.548	1.551
A – ring			
C5–C4	1.571	1.562	1.571
C4–C3	1.560	1.534	1.562
C3–C2	1.524	1.529	1.525
C2–C1	1.533	1.520	1.533
C1–C10	1.555	1.536	1.556
C24–H24a	1.096	1.072	1.096
C24–H24b	1.091	0.887	1.091
C24–H24c	1.094	0.985	1.093
C23–H23a	1.097	0.951	1.098
C23–H23b	1.094	0.895	1.094
C23–H23c	1.098	1.020	1.096
C3–H3	1.105	0.954	1.105

C3–O1	1.429	1.435	1.425
1O–H1	0.969	0.958	0.969
C2–H2a	1.096	0.926	1.096
C2–H2b	1.096	0.973	1.096
C1–H1a	1.100	0.890	1.100
C1–H1b	1.097	1.150	1.097
C4–C24	1.542	1.527	1.542
C4–C23	1.549	1.567	1.548

* The numbering of atoms in the ursolic acid molecule is shown in Figure 1a.

Table S2. The bond angles of ursolic acid and cation (+1) complex of Cu(II) molecules calculated using B3LYP/6-31G(d)*.

Bond Angles (°)			
	**UA calc.**	**UA exp. [33]**	**UA-Cu calc.**
O3–C28–O2	120.95	120.77	115.48
C28–O2–H2a	105.46	161.65	86.92
C28–O3–H2a	56.50	38.11	87.21
O2–H2a–O3	77.09	31.08	70.38
C28–C17–C16	111.38	109.35	110.83
C28–C17–C18	108.45	112.31	108.60
C28–C17–C22	105.86	102.86	104.83
O3–C28–C17	126.15	114.72	121.59
O2–C28–C17	112.79	124.21	122.83
E – ring			
C17–C22–C21	111.53	113.31	112.08
C17–C18–C19	110.00	114.04	109.41
C17–C18–C13	112.73	110.86	112.73
C17–C16–C15	112.35	111.96	112.58
C22–C21–C20	110.77	113.16	110.88
C22–C17–C18	110.99	111.21	111.10
C22–C17–C16	109.35	110.45	109.96
C21–C20–C19	112.01	102.04	112.31
C21–C20–C30	110.74	110.51	110.71
C20–C19–C29	110.00	109.68	110.24
C20–C19–C18	109.70	111.79	110.17
C30–C20–C19	111.24	112.75	110.73
C19–C18–C13	116.81	112.36	116.43
C29–C19–C18	113.16	111.05	112.98
C18–C17–C16	110.70	110.44	111.33
C18–**C13–C12**	117.52	119.39	116.92
C18–**C13**–C14	121.15	119.85	122.28
C17–C16–H16a	109.57	110.74	109.49
C17–C16–H16b	107.96	105.36	107.51
C17–C22–H22a	108.30	108.43	107.01
C17–C22–H22b	109.84	110.99	110.01
C17–C18–H18	106.47	111.54	107.60
H22a–C22–H22b	105.87	103.94	105.67
C22–C21–H21a	109.05	105.00	109.54
C22–C21–H21b	110.20	115.69	109.58
H22a–C22–C21	109.59	107.09	109.98

H22b–C22–C21	111.51	112.68	111.79
H21a–C21–H21b	106.27	102.59	106.08
H21a–C21–C20	110.59	110.70	110.81
H21b–C21–C20	109.86	108.77	109.83
C21–C20–H20	107.77	115.87	107.75
C20–C19–H19	107.39	101.71	107.67
C20–C30–H30a	110.93	94.27	110.67
C20–C30–H30b	111.09	98.44	111.26
C20–C30–H30c	112.05	105.81	112.11
H30a–C30–H30b	107.55	117.46	107.61
H30a–C30–H30c	107.59	119.75	107.51
H30b–C30–H30c	107.42	114.53	107.47
C30–C20–65H	107.75	106.43	107.96
C19–C18–H18	105.18	102.02	104.42
C19–C20–H20	107.10	101.81	107.18
59H–C19–C29	107.58	106,79	107.94
H19–C19–C18	108.82	115.30	107.66
C19–C29–H29a	110.87	107.34	111.20
C19–C29–H29b	110.80	103.91	110.20
C19–C29–H29c	112.15	114.72	112.44
H29b–C29–H29c	107.85	122.61	107.53
H29c–C29–H29a	107.39	106.61	107.94
H29a–C29–H29b	107.59	102.00	107.31
D – ring			
C13–C12–C11	126.19	126.31	125.99
C13–C14–C27	108.38	107.81	107.17
C13–C14–C15	108.91	111.14	109.43
C13–C14–C8	110.35	109.81	110.58
C14–C15–C16	113.83	114.98	114.16
C14–C8–C26	109.68	109.61	109.63
C14–C8–C7	110.07	111.13	110.23
C14–C8–C9	108.39	107.11	108.46
C15–C14–C27	107.09	106.29	106.89
C15–C14–C8	109.89	110.28	110.46
C13–C12–H12	118.55	126.49	118.19
C14–C27–H27a	110.87	107.66	111.28
C14–C27–H27b	111.83	116.03	110.86
C14–C27–H27c	112.45	118.04	112.48
C14–C15–H15a	109.26	106.01	108.94
C14–C15–H15b	110.42	107.95	110.38
H27a–C27–H27b	106.78	107.53	106.86
H27a–C27–H27c	106.60	91.88	106.84
H27b–C27–H27c	108.01	115.09	108.25
H15a–C15–H15b	106.33	96.93	106.16
H15a–C15–C16	109.30	116.93	109.15
H15b–C15–C16	107.42	112.13	107.74
C15–C16–H16a	109.73	105.46	109.78
C15–C16–H16b	110.66	105.01	110.96
H16a–C16–H16b	106.37	118.32	106.30
C – ring			
C12–C11–C9	113.11	112.85	114.12

C11–C9–C8	109.69	109.85	110.12
C11–C9–C10	113.64	114.10	113.28
C9–C8–C26	110.56	109.81	110.86
C9–C8–C14	108.39	107.11	108.46
C9–C8–C7	109.72	111.91	109.36
C9–C10–C25	113.54	113.46	113.51
C9–C10–C1	107.54	107.04	107.37
C9–C10–C5	106.61	105.67	106.32
C8–C14–C27	112.11	111.45	110.46
C8–C14–C15	109.89	110.28	110.46
C8–9C–C6	114.39	114.34	114.27
C8–C9–C10	117.94	117.48	117.90
H12–C12 –C11	115.25	107.19	115.82
C12–C11–H11a	108.17	104.42	107.41
C12–C11–H11b	108.61	110.45	108.08
H11b–C11–C9	110.16	102.98	110.81
H11b–C11–H11a	105.03	116.86	103.95
H11a–C11–C9	111.41	109.14	111.89
C1 –C9–H9	105.78	108.06	105.74
H9–C9–6C	102.97	101.09	102.92
H9–C9–C8	105.52	104.89	105.58
C8–C26–H26a	111.65	109.05	111.41
C8–C26–H26b	111.26	113.59	111.37
C8–C26–H26c	111.88	121.26	112.27
C8–C7–H7a	109.61	107.02	109.73
C8–C7–H7b	110.09	107.21	109.98
H26a–C26–H26b	106.55	94.94	106.40
H26a–C26–H26c	107.48	106.63	107.23
H26b–C26–H26c	107.77	107.96	107.89
	B – ring		
C7–C6–C5	109.99	109.75	110.43
C6–C5–C10	109.95	110.14	110.12
C6–C5–C4	115.17	114.94	115.08
C5–C10–C25	113.46	108.67	113.56
C5–C10–C9	106.61	105.67	106.32
C5–C10–C1	107.90	108.67	107.99
C5–C4–C24	114.88	115.04	114.92
C5–C4–C23	108.70	107.65	108.80
C –C4–C3	107.35	106.56	107.30
H7a–C7–H7b	106.27	106.58	106.36
H7–C7–C6	109.71	113.01	109.73
H7b–C7–C6	106.44	108.01	106.44
C7–C6–H6a	108.04	107.63	107.67
C7–C6–H6b	110.94	109.16	110.96
H6a–C6–H6b	105.87	110.77	105.78
H6a–C6–C5	110.79	107.63	110.61
H6b–C6–C5	111.11	109.16	111.23
C6–C5–H5	105.31	102.43	105.39
H5–C5–C4	103.34	106.62	103.46
C10–C1–H1b	110.22	114.83	110.35
C10–C1–H1a	108.89	111.94	108.92

C10–C25–H25a	111.39	115.50	111.96
C10–C25–H25b	111.18	111.72	111.04
C10–C25–H25c	113.52	121.04	113.51
C10–C5–H5	104.05	108.53	104.12
	A - ring		
C10–C1–C2	113.71	114.00	113.54
C1–C2–C3	111.83	109.86	111.99
C2–C3–C4	113.49	112.76	113.49
**C2–C3–O1**	106.38	108.36	106.22
C3–C4–C5	107.35	106.56	107.30
C3–C4–C24	111.21	112.71	111.08
C3–C4–C23	107.26	106.39	107.22
C24–C4–C23	107.18	108.05	107.26
C1–C2–H2a	111.51	116.11	111.53
C1–C2–H2b	109.93	113.67	109.77
H1a–C1–H1b	106.27	101.31	106.40
H1a–C1–C2	108.49	108.81	108.45
H1b–C1–C2	108.98	104.96	108.90
H2a–C2–H2b	105.94	106.33	105.87
H2a–C2–C3	109.13	109.36	109.12
H2b–C2–C3	108.28	100.61	108.32
C2–C3–H3	107.54	104.79	107.68
**H3–C3–O1**	109.24	108.03	109.53
H3–C3–C4	106.78	111.68	106.83
**C4–C3–O1**	113.23	110.92	112.97
**C3–1O–H1**	108.02	138.13	108.61
C4–C23–H23a	111.70	107.98	111.85
C4–C23–H23b	112.16	111.23	112.36
C4–C23–H23c	110.89	116.46	110.49
H23a–C23–H23b	107.93	109.41	108.01
H23a–C23–H23c	107.88	104.61	107.86
H23b–C23–H23c	106.00	106.82	105.99
C4–C24–H24b	113.31	120.30	113.48
C4–C24–H24c	109.83	117.84	109.64
C4–C24–H24a	110.79	111.24	110.97
H24a–C24–H24c	107.93	107.13	107.91
H24a –C24–H24b	107.82	95.41	107.81
H24b–C24–H24c	106.94	102.06	106.80

*The numbering of atoms in the ursolic acid molecule is shown in Figure 1a.

Table S3. Data of NBO atomic charge analysis for ursolic acid and cation (+1) complex of Cu(II).

**Atom**	**Charge (e^1)**	
	UA	Cu(II) UA
O2	-0.716	-0.694
O3	-0.605	-0.705
C31	0.848	0.858
H2a/Cu	0.497	1.141
C17	-0.146	-0.142
	E – ring	
C22	-0.443	-0.439
C21	-0.464	-0.469

C20	-0.248	-0.253
C19	-0.241	-0.232
C18	-0.267	-0.272
C30	-0.670	-0.675
C29	-0.666	-0.673
H22a	0.234	0.255
H22b	0.250	0.254
H21a	0.243	0.240
H21b	0.241	0.259
H30a	0.232	0.245
H30b	0.221	0.225
H30c	0.230	0.239
H29a	0.226	0.230
H29b	0.228	0.247
H29c	0.237	0.239
	D - ring	
C16	-0.450	-0.425
C15	-0.450	-0.453
C14	-0.060	-0.071
C13	-0.026	0.042
H13a	0.257	0.267
H13b	0.231	0.248
H15a	0.240	0.259
H15b	0.239	0.257
H18	0.269	0.274
	C–ring	
C12	-0.194	-0.103
C11	-0.477	-0.495
C9	-0.236	-0.240
C8	-0.036	0.035
C27	-0.666	-0.663
C27	-0.666	-0.663
C26	-0.684	-0.681
H20	0.226	0.235
H11b	0.247	0.274
H11a	0.238	0.259
H27a	0.232	0.234
H27b	0.228	0.245
H27c	0.231	0.242
H26a	0.234	0.249
H26b	0.228	0.233
H26c	0.243	0.219
H9	0.235	0.242
	B and A–ring	
C7	-0.445	-0.449
C6	-0.442	-0.444
C5	-0.250	-0.250
C10	-0.040	-0.041
C25	-0.673	-0.675
H7a	0.235	0.239
H7b	0.229	0.233

H6a	0.237	0.249
H6b	0.226	0.228
H25a	0.228	0.231
H25b	0.232	0.224
H25c	0.229	0.242
O1	-0.755	-0.750
H1	0.471	0.478
C1	-0.453	-0.454
C2	-0.470	-0.472
C3	0.113	0.112
C4	-0.075	-0.075
C24	0.675	0.678
C23	-0.671	-0.674
H1a	0.241	0.239
H1b	0.228	0.229
H2a	0.232	0.236
H2b	0.251	0.259
H24b	0.230	0.229
H24a	0.229	0.229
H24c	0.239	0.250
H23b	0.237	0.238
H23c	0.217	0.227
H23a	0.229	0.229
H5	0.231	0.232

*The numbering of atoms rings in the ursolic acid molecule is shown in Figure 1a.
